# Reconstituted HDL (Milano) Treatment Efficaciously Reverses Heart Failure with Preserved Ejection Fraction in Mice

**DOI:** 10.3390/ijms19113399

**Published:** 2018-10-30

**Authors:** Mudit Mishra, Ilayaraja Muthuramu, Joseph Pierre Aboumsallem, Herman Kempen, Bart De Geest

**Affiliations:** 1 Centre for Molecular and Vascular Biology, Department of Cardiovascular Sciences, Catholic University of Leuven, 3000 Leuven, Belgium; mudit.mishra@kuleuven.be (M.M.); ilayaraja.muthuramu@kuleuven.be (I.M.); josephpierre.aboumsallem@kuleuven.be (J.P.A.); 2 The Medicines Company (Schweiz), CH-8001 GmbH Zürich, Switzerland; hermankempen@gmail.com

**Keywords:** Reconstituted HDL, cardiac hypertrophy, coconut oil, heart failure, HFpEF, cardiac function, exercise capacity, lactate, acetyl-coenzyme A carboxylase, transforming growth factor-β1

## Abstract

Heart failure with preserved ejection fraction (HFpEF) represents a major unmet therapeutic need. This study investigated whether feeding coconut oil (CC diet) for 26 weeks in female C57BL/6N mice induces HFpEF and evaluated the effect of reconstituted high-density lipoprotein (HDL)_Milano_ (MDCO-216) administration on established HFpEF. Eight intraperitoneal injections of MDCO-216 (100 mg/kg protein concentration) or of an equivalent volume of control buffer were executed with a 48-h interval starting at 26 weeks after the initiation of the diet. Feeding the CC diet for 26 weeks induced pathological left ventricular hypertrophy characterized by a 17.1% (*p* < 0.0001) lower myocardial capillary density and markedly (*p* < 0.0001) increased interstitial fibrosis compared to standard chow (SC) diet mice. Parameters of systolic and diastolic function were significantly impaired in CC diet mice resulting in a reduced stroke volume, decreased cardiac output, and impaired ventriculo-arterial coupling. However, ejection fraction was preserved. Administration of MDCO-216 in CC diet mice reduced cardiac hypertrophy, increased capillary density (*p* < 0.01), and reduced interstitial fibrosis (*p* < 0.01). MDCO-216 treatment completely normalized cardiac function, lowered myocardial acetyl-coenzyme A carboxylase levels, and decreased myocardial transforming growth factor-β1 in CC diet mice. In conclusion, the CC diet induced HFpEF. Reconstituted HDL_Milano_ reversed pathological remodeling and functional cardiac abnormalities.

## 1. Introduction

Heart failure is the chronic, progressive condition in which cardiac dysfunction is responsible for failure of the heart to pump blood at a rate commensurate with tissue requirements for oxygen and metabolic substrates. Symptoms and signs of heart failure include dyspnoea, fatigue, reduced exercise tolerance, and fluid accumulation. Prevalence of heart failure can be estimated at 2% in the Western world whereas incidence approaches 5–10 per 1000 persons per year [1]. However, the prevalence and incidence increase progressively with age [2,3]. The prevalence of heart failure is 7% in the age group 75–84 years and over 10% in those older than 85 years [4]. The 5-year age-adjusted mortality rates after onset of heart failure are 50% in men and 46% in women [5]. Taken together, this syndrome is the cardiovascular epidemic of this century [6].

Approximately 50% of patients with clinical features of chronic heart failure suffer from heart failure with reduced ejection fraction (HFrEF) and 50% have heart failure with preserved ejection fraction (HFpEF) [7]. HFpEF is a clinical syndrome that is characterized by classical heart failure symptoms with increased left ventricular filling pressure and an ejection fraction equal to or greater than 50%. As the population ages, HFpEF will continue to be a growing public health problem [7]. The large majority of clinical heart failure trials have been focused on patients with HFrEF. Inhibition of the renin-angiotensin-aldosterone system and of the sympathetic nervous system both improve survival and decrease hospitalizations in patients with HFrEF. In contrast to these prominent advances in the treatment of HFrEF, drug strategies with strong evidence in HFrEF have proved unsuccessful in HFpEF and mortality in HFpEF patients has remained unchanged.

HFpEF was historically referred to as ‘diastolic heart failure’. Patients exhibit decreased left ventricular active relaxation and/or increased passive left ventricular diastolic stiffness at rest [8]. Rather than being solely caused by diastolic dysfunction, HFpEF is the manifestation of reduced ventricular diastolic reserve function, impaired ventricular systolic reserve function, decreased heart rate reserve, atrial dysfunction, pulmonary hypertension, and abnormalities in skeletal muscle [9]. Many of these abnormalities are not present at rest but become apparent during exercise (reserve limitation) [9].

Observational studies support an independent inverse association between high-density lipoprotein (HDL)-cholesterol levels and heart failure incidence. In Framingham Heart Study subjects free of coronary heart disease at baseline, lower HDL-cholesterol levels were independently associated with increased heart failure incidence after adjustment for interim myocardial infarction and clinical covariables [10]. HDL are circulating multimolecular platforms that exert divergent functions and pleiotropic properties of HDL may exert beneficial effects on the myocardium [11,12,13,14]. Continuous infusion of HDL has been shown to inhibit cardiac hypertrophy in vivo [15,16]. HDL-raising adeno-associated viral serotype 8-human apolipoprotein (apo) A-I gene therapy in mice exerted anti-hypertrophic effects on the myocardium under conditions of pressure overload and counteracted pathological remodeling as evidenced by increased capillary density and reduced myocardial fibrosis [17]. Furthermore, cardiac function was improved and heart failure was prevented following HDL-raising apo A-I gene transfer [17]. In contrast, dysfunctional HDL in scavenger receptor class B, type I (SR-BI) deficient mice led to more pronounced cardiac hypertrophy, to more severe pathological remodeling, to more prominent cardiac dysfunction, and to heart failure in mice with pressure overload [18].

MDCO-216 is a form of reconstituted HDL that contains highly purified recombinant dimeric apoA-I_Milano_ complexed with 1-palmitoyl-2-oleoyl-sn-glycero-3-phosphatidylcholine (POPC) [19]. The clinical safety of MDCO-216 has been established in several studies [19,20,21,22]. We have recently demonstrated that intervention with MDCO-216 reverses established pathological remodeling induced by pressure overload and constitutes a successful treatment for heart failure in mice with pressure overload [23]. Since pressure overload induced by transverse aortic constriction (TAC) leads to cardiac dilatation and HFrEF [24], the question remains whether this HDL-targeted intervention may be effective in HFpEF. 

The objective of the current study was twofold. First, we intended to investigate whether prolonged feeding of a 0.2% cholesterol 10% coconut oil diet induces HFpEF in female C57BL/6N mice. This hypothesis was elicited by our previous findings that feeding this diet for 13 weeks induced myocardial fibrosis and diastolic dysfunction in female C57BL/6N mice without inducing cardiac steatosis [25]. Secondly, we examined the hypothesis that intervention with MDCO-216 in mice with established HFpEF may reverse cardiac dysfunction and pathological remodeling.

## 2. Results

### 2.1. The 0.2% Cholesterol 10% Coconut Oil (CC) Diet Induces Cardiac Hypertrophy, Reduces Myocardial Capillary Density, and Increases Myocardial Fibrosis

**Figure 1:**
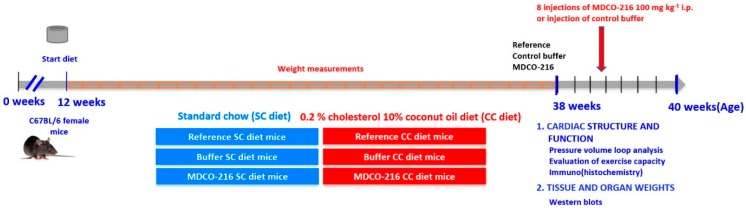
Schematic representation of the study design.

**Figure 2:**
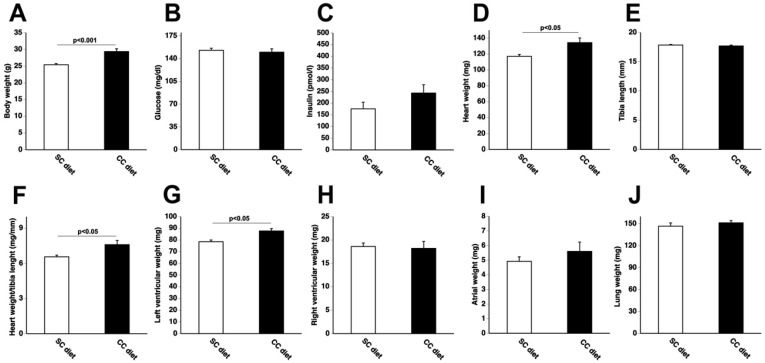
Body weight (**A**), glucose level (**B**), insulin (**C**), heart weight (**D**), tibia length (**E**), heart weight/tibia length (**F**), left ventricular weight (**G**), right ventricular weight (**H**), atrial weight (**I**), and lung weight (**J**) in C57BL/6 standard chow (SC) diet mice and in C57BL/6 coconut oil (CC) diet mice. CC diet was initiated at 12 weeks of age. Quantifications were performed at 38 weeks, 26 weeks after the start of the diet. SC diet mice and CC diet mice are indicated by open bars and closed bars, respectively. All data represent means ± SEM (*n* = 15).

**Figure 3:**
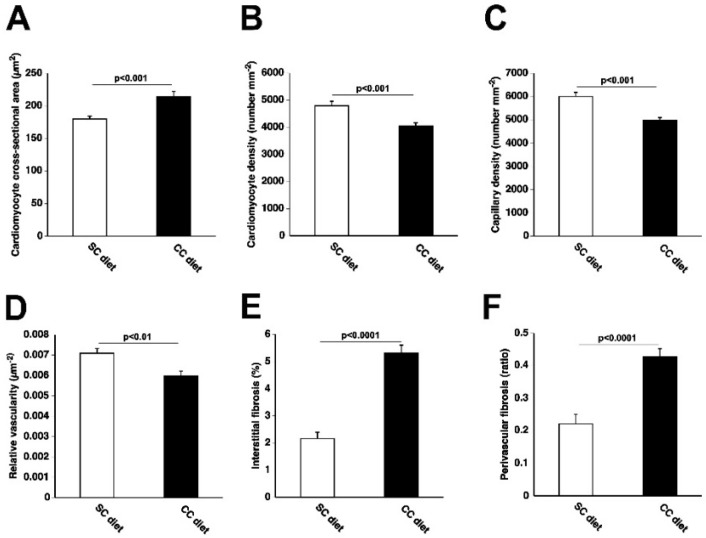
Quantification of histological and immunohistochemical parameters in the myocardium of C57BL/6 SC diet mice and C57BL/6 CC diet mice. Bar graphs showing the cardiomyocyte cross-sectional area (**A**), cardiomyocyte density (**B**), capillary density (**C**), relative vascularity (**D**), interstitial fibrosis (**E**), and perivascular fibrosis (**F**) in SC diet mice (*n* = 21) and CC diet mice (*n* = 30) at 38 weeks, 26 weeks after the start of diet. SC diet mice and CC diet mice are indicated by open bars and closed bars, respectively. All data represent means ± SEM.

The global study design is illustrated in Figure 1. The 0.2% cholesterol 10% coconut oil diet (CC diet) was initiated at 12 weeks of age in female C57BL/6N mice. Quantifications in the standard chow (SC) diet and CC diet groups were performed at the age of 38 weeks. Body weight after 26 weeks of feeding the CC diet was moderately (1.15-fold (*p* < 0.001)) higher than in the SC diet group (Figure 2A). No significant differences in blood glucose levels (Figure 2B) or in plasma insulin levels (Figure 2C) were observed. Heart weight (Figure 2D) and heart weight/tibia length ratio (Figure 2F) in CC diet mice were 1.15-fold (*p* < 0.05) and 1.16-fold (*p* < 0.05) higher, respectively, than in SC diet mice indicating cardiac hypertrophy. Left ventricular weight (Figure 2G) was significantly (*p* < 0.05) higher in CC diet mice than in SC diet mice whereas no significant differences of right ventricular weight (Figure 2H), atrial weight (Figure 2I), and lung weight (Figure 2J) were observed. At the microscopic level, cardiomyocyte cross-sectional area was 1.19-fold (*p* < 0.001) larger in CC diet mice than in SC diet mice (Figure 3A). Cardiomyocyte hypertrophy was paralleled by a decrease (*p* < 0.001) of cardiomyocyte density (Figure 3B). Capillary density was 17.1% (*p* < 0.0001) lower in CC diet mice than in standard chow mice (Figure 3C). Furthermore, relative vascularity (Figure 3D) was significantly (*p* < 0.01) reduced and interstitial fibrosis (Figure 3E) was strongly increased (*p* < 0.0001) in CC diet mice. The degree of perivascular fibrosis was 1.93-fold (*p* < 0.0001) higher in CC diet mice than in SC diet mice (Figure 3F). Taken together, the CC diet causes cardiac hypertrophy and cardiomyocyte hypertrophy. Cardiac hypertrophy is pathological as evidenced by the reduced capillary density and the increased interstitial and perivascular fibrosis.

### 2.2. Hemodynamic Deterioration in CC Diet Mice Is Consistent with Heart Failure with Preserved Ejection Fraction

Hemodynamic data in female C57BL/6N mice fed the SC diet and in C57BL/6N mice fed the CC diet were generated using the Millar Pressure-Volume (PV) Loop System (MPVS) and are summarized in Table 1. The CC diet induced both systolic and diastolic dysfunction. Preload recruitable stroke work (PRSW), the slope of the relationship between end-diastolic volume (EDV) and stroke work, and end-systolic elastance (E_es_), the slope of the end-systolic pressure-volume relationship (ESPVR), are load-independent parameters of left ventricular contractility. PRSW was reduced by 20.9% (*p* < 0.05) in the CC diet mice compared to SC diet mice. E_es_ was 53.2% (*p* < 0.0001) lower in CC diet mice than in SC diet mice. The effective arterial elastance (E_a_) was similar in both groups, reflecting a proportional reduction of end-sytolic pressure (P_es_) and stroke volume in the CC diet group. The E_a_/E_es_ ratio was significantly (*p* < 0.0001) increased in CC diet mice, indicating impaired ventriculo-arterial coupling. The slope of the end-diastolic pressure volume relationship (EDPVR), reflecting the elastance or inverse of compliance of the left ventricular myocardium during the filling phase, was significantly (*p* < 0.05) increased in CC diet mice compared to SC diet mice (Table 1). The absolute value of dP/dt_min_ was significantly lower (*p* < 0.05) in the CC diet mice than in SC diet mice whereas the time constant of isovolumetric relaxation, tau, was significantly increased (*p* < 0.001) in CC diet mice.

Stroke volume (*p* < 0.05) and cardiac output (*p* < 0.05) were lower in CC diet mice than in SC diet mice (Table 1). This occurred in the absence of left ventricular dilatation and in fact, EDV tended to decrease. The peak filling rate (dV/dt_max_) (*p* < 0.05) and the peak emptying rate (dV/dt_min_) (*p* < 0.01) were significantly reduced in CC diet mice compared to SC diet mice. Ejection fraction in CC diet mice was not significantly reduced and remained clearly above 50% (Table 1). Taken together, deterioration of hemodynamic function in CC diet mice is consistent with heart failure with HFpEF. 

### 2.3. Reconstituted HDL_Milano_ Significantly Decreases Cardiac Hypertrophy, Increases Myocardial Capillary Density, and Decreases Myocardial Fibrosis in CC Diet Mice

Reference SC diet mice and reference CC diet mice were analyzed at the age of 38 weeks. MDCO-216 SC diet and MDCO-216 CC diet intervention groups were treated with 8 intraperitoneal administrations of 100 mg/kg (protein concentration) of MDCO-216 at an interval of 48 h each starting at the age of 38 weeks. Control buffer SC diet and control buffer CC diet mice were injected with the same volume of buffer solution.

Total cholesterol, non-HDL cholesterol, and HDL cholesterol plasma levels in C57BL/6 mice at time of sacrifice are represented in Table 2. No significant differences of total cholesterol, HDL cholesterol, and non-HDL cholesterol were observed. Murine apo A-I levels in MDCO-216 CC diet mice (31.5 ± 3.0 mg/dL; *n* = 5) were reduced by 70.1% and by 68.0% compared to reference CC diet mice (105 ± 6 mg/dL; *n* = 5) and buffer CC diet mice (98.3 ± 7.4 mg/dL; *n* = 5), respectively.

**Figure 4:**
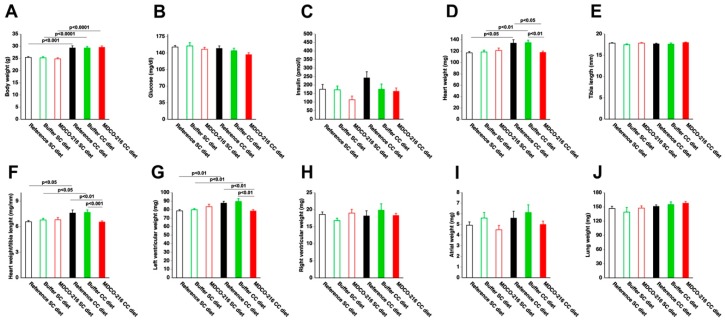
Body weight (**A**), glucose level (**B**), insulin (**C**), heart weight (**D**), tibia length (**E**), heart weight/tibia length (**F**), left ventricular weight (**G**), right ventricular weight (**H**), atrial weight (**I**), and lung weight (**J**) in C57BL/6 SC diet mice and in C57BL/6 CC diet mice. CC diet was initiated at 12 weeks of age. Quantifications were performed at 38 weeks (reference mice) or at 40 weeks (buffer mice and MDCO-216 mice) on SC diet or CC diet. SC diet mice and CC diet mice are indicated by open bars and closed bars, respectively. All data represent means ± SEM (*n* = 15).

**Figure 5:**
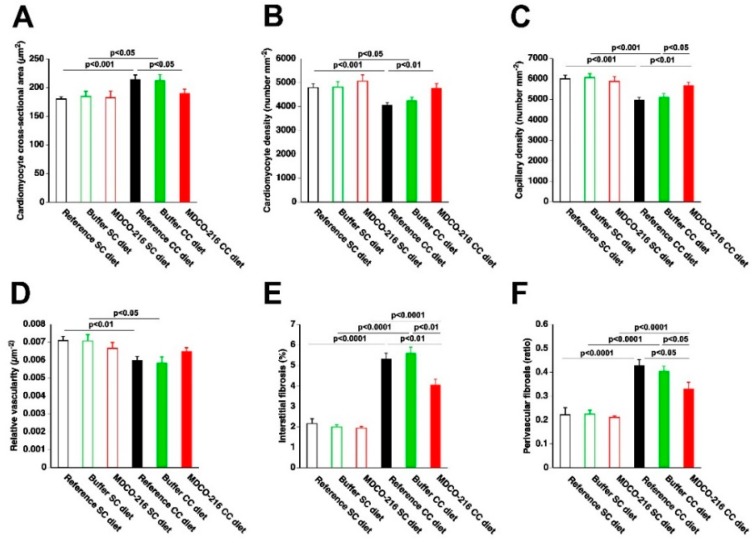
Quantification of histological and immunohistochemical parameters in the myocardium of C57BL/6 SC diet mice and C57BL/6 CC diet mice. Bar graphs showing the cardiomyocyte cross-sectional area (**A**), cardiomyocyte density (**B**), capillary density (**C**), relative vascularity (**D**), interstitial fibrosis(**E**), and perivascular fibrosis (**F**) in reference SC diet mice (*n* = 21), buffer SC diet mice (*n* = 12), MDCO-216 SC diet mice (*n* = 12), reference CC diet mice (*n* = 30), buffer CC diet mice (*n* = 14), and MDCO-216 CC diet mice (*n* = 17) at 38 weeks (reference mice) and at 40 weeks (buffer mice and MDCO-216 mice). The CC diet was initiated at 12 weeks of age. SC diet mice and CC diet mice are indicated by open bars and closed bars, respectively. All data represent means ± SEM.

**Figure 6:**
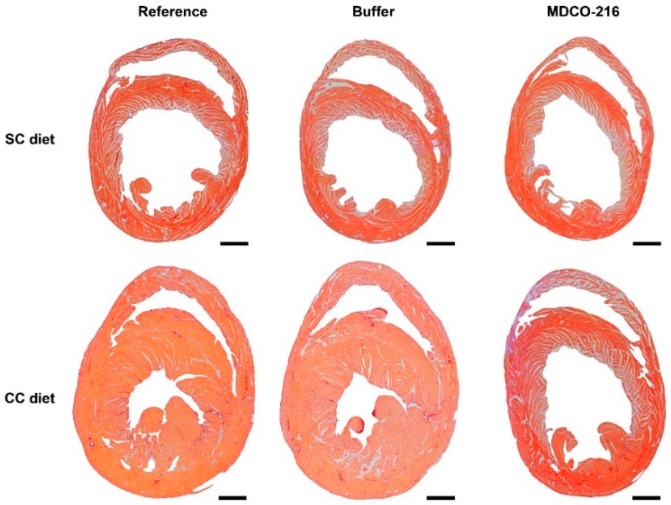
Representative Sirius-red stained cross-sections of reference, buffer, and MDCO-216 SC diet and CC diet mice. Scale bar represents 1 mm.

**Figure 7:**
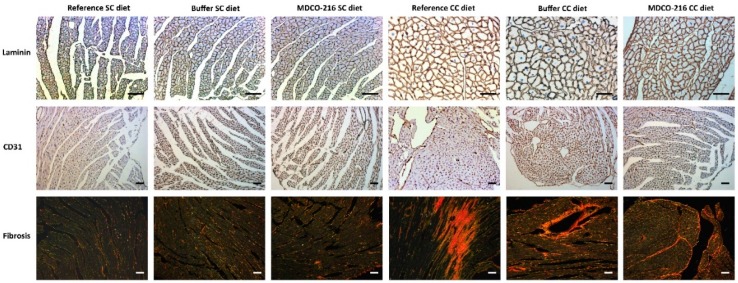
Immunohistochemical and histochemical analysis of the myocardium of reference, buffer, and MDCO-216 SC diet and CC diet mice. Representative photomicrographs show laminin-stained cardiomyocytes, CD31-positive capillaries, and Sirius-red-stained collagen. Scale bar represents 50 µm.

Body weight (Figure 4A) was moderately increased in all three CC diet groups compared to the respective SC diet groups. No significant differences of glucose levels were observed (Figure 4B). Insulin levels were not significantly increased in CC diet groups compared to respective SC diet groups (Figure 4C). Heart weight in MDCO-216 CC diet mice was 12.1% (*p* < 0.05) and 12.6% (*p* < 0.01) lower than in reference CC diet mice and buffer CC diet mice, respectively (Figure 4D). Furthermore, heart weight in MDCO-216 CC diet mice was not significantly different compared to MDCO-216 SC diet mice. Similar differences were observed for heart weight/tibia length ratio (Figure 4F). Left ventricular weight was reduced by 10.6% (*p* < 0.01) and 12.5% (*p* < 0.01) in MDCO-216 CC diet mice compared to reference CC diet mice and buffer CC diet mice, respectively (Figure 4G). Right ventricular weight (Figure 4H), atrial weight (Figure 4I), and lung weight (Figure 4J) were not significantly different. At the histological level, cardiomyocyte cross-sectional area was reduced by 11.5% (*p* < 0.05) in MDCO-216 CC diet mice compared to reference CC diet mice (Figure 5A). This was paralleled by a significant (*p* < 0.01) increase of cardiomyocyte density (Figure 5B). Capillary density in MDCO-216 CC diet mice was 1.14-fold (*p* < 0.01) and 1.11-fold (*p* < 0.05) higher than in reference CC diet mice and in buffer CC diet mice, respectively (Figure 5C). The increase of relative vascularity in MDCO-216 CC diet mice compared to the other two CC diet groups did not reach statistical significance (Figure 5D). However, the degree of interstitial fibrosis (Figure 5E) and of perivascular fibrosis (Figure 5F) was significantly lower in MDCO-216 CC diet mice compared to both reference CC diet mice and buffer CC diet mice. All in all, intervention with MDCO-216 in CC diet reverses cardiac hypertrophy and cardiomyocyte hypertrophy and mitigates features of pathological remodeling as evidenced by the increased capillary density and the reduced interstitial and perivascular fibrosis. As expected, MDCO-216 did not result in any effect in SC diet mice. (Figure 4 and Figure 5). Representative Sirius red-stained cross-sections of hearts of all SC diet and CC diet groups are shown in Figure 6. Representative photomicrographs illustrating laminin-stained cardiomyocytes, CD31-positive capillaries, and Sirius red-stained collagen viewed under polarized light in the three SC diet groups and in the three CC diet groups are shown in Figure 7. 

### 2.4. Hemodynamic Function Is Restored in CC Diet Mice following Intervention with MDCO-216

An overview of hemodynamic data in reference, buffer, and MDCO-216 SC diet and CC diet mice is provided in Table 3. As expected, MDCO-216 had no effect in SC diet mice that are characterized by a normal cardiac function. Intervention with buffer in CC diet mice did not have any beneficial effect on hemodynamic parameters and a similar pattern of abnormalities was present as observed in reference CC diet mice. In contrast, intervention with MDCO-216 completely reversed systolic (dP/dt_max_, PRSW, E_es_, dV/dt_min_) and diastolic abnormalities (dP/dt_min_, tau, slope EDPVR, dV/dt_max_). Stroke volume, cardiac output, and ventriculo-arterial coupling were normalized (Table 3). Taken together, abnormalities in hemodynamic parameters in reference CC diet mice and in buffer CC diet mice that are consistent with HFpEF are completely reversed in MDCO-216 CC diet mice.

### 2.5. MDCO-216 Normalizes Myocardial Acetyl-Coenzyme A (Acetyl-CoA) Carboxylase (ACC) Levels and DECREASES Myocardial Transforming Growth Factor (TGF)-β1 Levels in CC Diet Mice

**Figure 8:**
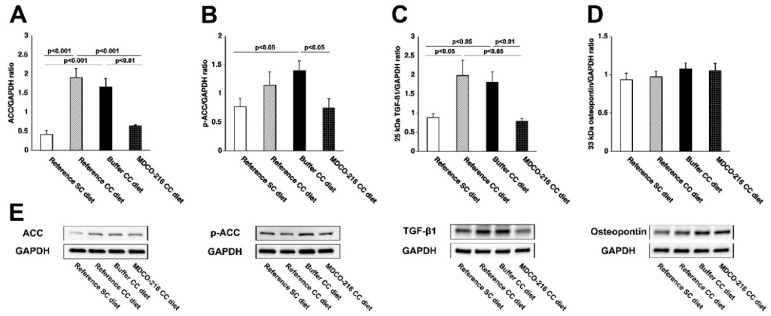
Quantification of proteins in the myocardium by western blot. Bar graphs illustrating ACC (**A**), p-ACC (**B**), TGF-β1 (**C**), and osteopontin (**D**) protein levels quantified by western blot in the myocardium. SC diet mice and CC diet mice are indicated by open bars and closed bars, respectively. Representative images of western blots are shown in panel E. All protein levels were normalized to the glyceraldehyde-3-phosphate dehydrogenase (GAPDH) protein level. All data represent means ± SEM (*n* = 6).

Myocardial levels of the key metabolic enzyme acetyl-coenzyme A (acetyl-CoA) carboxylase (ACC) were 4.64-fold (*p* < 0.001) and 4.02-fold (*p* < 0.001) higher in reference CC diet mice and in buffer CC diet mice, respectively, than in the respective SC diet groups (Figure 8A). However, no significant elevation of ACC was observed in MDCO-216 CC diet mice (Figure 8A). Myocardial levels of ACC in MDCO-216 CC diet mice were 66.0% (*p* < 0.001) and 60.8% (*p* < 0.01) lower than in reference CC diet mice and in buffer CC diet mice, respectively. Myocardial levels of p-ACC (Figure 8B), which constitutes the inactive form of the enzyme, were 45.9% (*p* < 0.05) lower in MDCO-216 CC diet mice compared to buffer CC diet mice. The 25-kDa isoform of TGF-β1 was increased by 2.24-fold (*p* < 0.05) and by 2.03-fold (*p* < 0.05) in reference CC diet mice and buffer CC diet mice, respectively, compared to respective SC diet groups (Figure 8C). Myocardial levels of the 25-kDa isoform of TGF-β1 in MDCO-216 CC diet mice were 60.2% (*p* < 0.05) and 56.2% (*p* < 0.01) lower than in reference CC diet mice and in buffer CC diet mice, respectively. No significant differences of the 33-kDa isoform of osteopontin were observed between different groups (Figure 8D). Similarly, no significant differences of the 44-kDa, 50-kDa, and 72-kDa isoforms of osteopontin were observed.

### 2.6. MDCO-216 Significantly Improves Exercise Capacity in CC DIET mice

**Figure 9:**
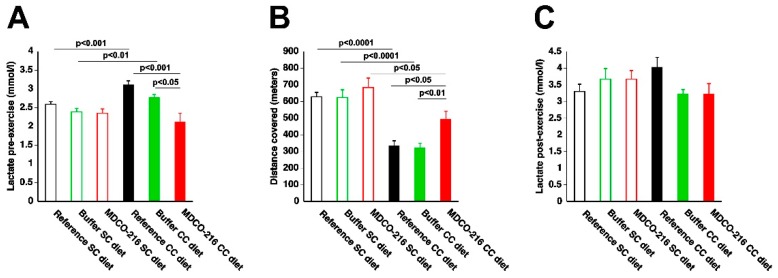
Lactate pre-exercise (**A**), distance covered (**B**), and lactate post-exercise (**C**) in C57BL/6 SC diet mice and in C57BL/6 CC diet mice. SC diet mice and CC diet mice are indicated by open bars and closed bars, respectively. All data represent means ± SEM (*n* = 15).

Exercise treadmill testing was applied to evaluate exercise capacity in the three SC diet and in the three CC diet groups. Lactate levels pre-exercise were 1.20-fold (*p* < 0.001) and 1.16-fold (*p* < 0.01) higher in reference CC diet mice and buffer CC diet mice, respectively, than in respective SC diet groups (Figure 9A). In contrast, lactate level pre-exercise was not increased in MDCO-216 CC diet mice compared to MDCO-216 SC diet mice and was 32.0% (*p* < 0.001) lower than in reference CC diet mice and 23.8% (*p* < 0.01) lower than in buffer CC diet mice (Figure 9A). The distance covered during exercise treadmill testing was reduced by 46.8% (*p* < 0.0001) and by 48.6% (*p* < 0.0001) in reference CC diet mice and in buffer CC diet mice, respectively, compared to respective SC diet groups (Figure 9B). The distance covered in Milano CC diet mice was 1.47-fold (*p* < 0.05) higher than in reference CC diet mice and 1.54-fold (*p* < 0.01) higher than in buffer CC diet mice. Lactate post-exercise levels were not different between different groups (Figure 9C). Taken together, the CC diet reduces exercise capacity. Treatment with MDCO-216 significantly improves exercise capacity in CC diet mice.

## 3. Discussion

The main findings of the present study are that: (1) feeding the CC diet for 26 weeks induced cardiac hypertrophy, pathological remodeling of the heart, and a compromised cardiac function consistent with abnormalities observed in HFpEF; (2) intervention with MDCO-216 in CC diet mice for two weeks reverse cardiac hypertrophy and pathological remodeling and successfully restored cardiac function; (3) MDCO-216 normalized myocardial levels of the key metabolic enzyme (ACC) and decreases myocardial levels of TGF-β1 in CC diet mice; and (4) MDCO-216 significantly improves exercise capacity in CC diet mice. Taken together, intervention with reconstituted HDL_Milano_ constitutes a successful treatment for HFpEF in mice. 

A major strength of the current study is that pressure-volume loop analysis was performed to analyze cardiac function. Cardiovascular function analysis should take into consideration the interaction between the left ventricle and the arterial system (ventriculo–arterial coupling). Effective arterial elastance (E_a_) is a measure of the net arterial load exerted on the left ventricle with a resistive and stiffness component and describes the ability of the vessel to accommodate pulsatile flow. Left ventricular end-systolic elastance (E_es_) is the slope parameter of the end-systolic pressure (ESP)-volume (ESV) relation (ESPVR) and is an index of left ventricular contractility. The ratio of E_a_ to E_es_ is a measure of the interaction of the left ventricle with the arterial system and is termed ventriculo–arterial coupling ratio. It represents a measure of pump efficiency in expelling blood into the vasulature and the most favorable ventriculo–arterial coupling occurs when the E_a_/E_es_ ratio lies in the range of 0.5–1.0 [26,27]. In this study, we show that the CC diet results in an unfavorably high E_a_/E_es_ ratio, which is completely normalized by treatment with MDCO-216. Furthermore, the CC diet did not result in left ventricular dilatation as evidenced by the complete absence of an increase of the end-diastolic volume (EDV) and ejection fraction was preserved. The systolic and diastolic function resulted in a significant decrease of stroke volume and cardiac output in CC diet mice, which was completely reversed by intervention with MDCO-216. 

Cardiac dysfunction in itself is not sufficient to make a diagnosis of heart failure. The presence of increased wet lung weight indicating pulmonary congestion is one clinical sign indicating the presence of heart failure in mice. No increased lung weight was observed in the current model. However, a diagnosis of heart failure is supported by increased lactate levels at rest in the reference CC diet mice and in the buffer CC diet mice. Increased lactate levels are observed in a subset of patients with acute heart failure [28] and of patients with advanced chronic heart failure [29]. Under physiological conditions, lactate at rest is in homeostatic balance with pyruvate, which is the final product of glycolysis [30]. A disproportionate increase in lactate concentration reflects, provided that lactate clearance is not affected, an increase of anaerobic glycolysis. The increased lactate in the reference CC diet mice and in the buffer CC diet mice indicates that the reduced cardiac output in these mice at rest failed to meet tissue requirements for oxygen. Therefore, increased lactate levels in these mice support the presence of heart failure. A second argument for the presence of heart failure in reference CC diet mice and in buffer CC diet mice is reduced exercise tolerance. Importantly, intervention with MDCO-216 in CC diet mice normalized lactate levels at rest and markedly improved exercise capacity. Reasons why exercise capacity was not fully restored in MDCO-216 CC diet mice compared to SC diet mice include the higher body weight in the former and potentially also the suboptimal cardiac function during exercise (reserve limitation) [9]. Reserve limitation may be the consequence of reduced ventricular diastolic reserve function and impaired ventricular systolic reserve function [9].

Administration of MDCO-216 in SC diet mice or in CC diet mice had no effect on HDL cholesterol levels 24 h after the last injection. Murine apo A-I levels were decreased following MDCO-216 administration, which has previously also been observed following MDCO-216 administration injection in another murine model [23] and after apo A-I_Milano_ gene transfer in mice [31]. Furthermore, HDL cholesterol in scavenger receptor class B, type I (SR-BI) deficient mice are prominently increased [18]. However, these mice are characterized by cardiac dysfunction both in the presence and absence of pressure overload, which is likely due to the dysfunctional nature of HDL [18]. Therefore, the effect of MDCO-216 on cardiac structure, remodeling, and function should be understood in terms of global alterations of the HDL proteome and lipidome, which affect HDL function. 

The global data on myocardial acetyl-CoA carboxylase (ACC) protein levels and on p-AAC proteins levels, representing the inactive form of the enzyme, suggest lower activity of ACC in MDCO-216 CC diet mice compared to reference CC diet mice and buffer CC diet mice. Because malonyl-CoA produced by acetyl-CoA carboxylase inhibits fatty acid transport across the mitochondrial membrane via carnitine palmitoyl transferase I [32], reduced ACC activity following MDCO-216 would maintain fatty acid oxidation and preserve metabolic flexibility. The effect of MDCO-216 on this key metabolic enzyme may be of particular importance. Cardiac-specific deletion of acetyl-CoA carboxylase 2, which induced a significant reduction of cardiac malonyl-CoA levels and led to a maintenance of fatty acid oxidation, has previously been shown to attenuate cardiac hypertrophy and to reduce cardiac fibrosis in a model of pressure overload [33].

TGF-β1 was markedly lower in MDCO-216 CC diet mice than in reference CC diet mice and buffer CC diet mice. This cytokine can promote cardiomyocyte apoptosis and cardiac hypertrophy and is a key player in myocardial fibrosis [34]. Moreover, HDL has been demonstrated to reduce TGF-β1-induced collagen deposition in murine fibroblasts [35] and to decrease TGF-β1-induced endothelial-mesenchymal transition in aortic endothelial cells in vitro [36]. Regression of myocardial fibrosis after administration of MDCO-216 has previously also been observed in mice with pressure overload [23]. 

Osteopontin is a secreted extracellular matrix-associated protein, with diverse biological activities. It is expressed as a 33 kDa nascent protein. Because of varying degrees of post-translational modifications, including glycosylation and phosphorylation, the molecular weight of osteopontin may range from 44 kDa to 75 kDa [37,38]. These post-translational modifications contribute to different functional activities of osteopontin. Cardiac hypertrophy following chronic pressure overload induced by aortic banding and angiotensin II-induced myocardial fibrosis are less pronounced in osteopontin-deficient mice [39,40]. In the current study, no significant alterations of different isoforms of osteopontin were observed.

A limitation of the current study is that the kinetics of apo A-I_Milano_ plasma levels following intraperitoneal administration and the distribution of apo A-I_Milano_ among different lipoproteins were not evaluated. A further limitation is that the effect of MDCO-216 was not contrasted with reconstituted HDL containing wild-type human apo A-I in a direct comparative study. Whether the effect of MDCO-216 is superior compared to reconstituted HDL containing wild-type apo A-I may require a very large study for reasons of statistical power.

In conclusion, this study demonstrates that feeding a 0.2% cholesterol 10% coconut oil diet induced cardiac hypertrophy, pathological remodeling of the heart, a compromised cardiac function with ejection fraction above 50%, and decreased exercise capacity. In this murine model of HFpEF, intervention with MDCO-216 in CC diet mice for two weeks reversed cardiac hypertrophy and pathological remodelling, successfully restored cardiac function, and improved exercise capacity. Intervention with reconstituted HDL_Milano_ constitutes a successful treatment for HFpEF in mice.

## 4. Materials and Methods

### 4.1. Reconstituted HDL_Milano_

MDCO-216 is a 1:1 by weight complex of recombinant dimeric apo A-I_Milano_ and 1-palmitoyl-2-oleoyl-sn-glycero-3-phosphatidylcholine (POPC). It was provided by The Medicines Company (Parsipanny, NJ, USA) as a solution in buffer containing mannitol 43.6 mM, sucrose 181 mM, NaH_2_PO_4_·2H_2_O 3.46 mM, and 8.43 mM Na_2_HPO_4_·7H_2_O.

The production of the recombinant protein in E. coli and its purification has been described in detail by Caparon et al. [41]. Briefly, complexation of dimeric apo A-I_Milano_ with POPC was performed using a high-pressure homogenization procedure. The final product contained 15 mg/mL protein, 15 mg/mL POPC, 1.3 mg/mL Na_2_HPO_4_·7H_2_O, 0.178 mg/mL NaH_2_PO_4_·2H_2_O, 62 mg/mL sucrose, and 8.2 mg/mL mannitol. This product had an osmolarity of 287 mOsmol/kg and a pH of 7.5. Characterization of the product by gel permeation chromatography showed that 89% of protein and phospholipid was recovered in a single peak of apparent molecular weight of around 150 kDa. Non-denaturing polyacrylamide gradient-gel electrophoresis revealed that the majority of the drug product displayed an apparent diameter of 8 nm and no free apoA-I_Milano_ was observed [42].

### 4.2. In Vivo Experiments and Study Design

All experimental procedures in animals were executed in accordance with protocols approved by the Institutional Animal Care and Research Advisory Committee of the Catholic University of Leuven (Approval number: P191/2015). C57BL/6N mice, originally purchased from Taconic (Ry, Denmark), were locally bred at the semi-specific pathogen free facility of the Catholic University of Leuven at Gasthuisberg. The study design is illustrated in Figure 1. All experimental mice were female and were fed standard chow (SC) diet (Sniff Spezialdiäten GMBH, Soest, Germany) or were fed standard chow diet supplemented with 0.2% cholesterol 10% coconut oil (CC diet). For preparation of the CC diet, 500 mL of coconut oil (91.4 g/100 mL; 3607 kJ/100 mL) (Sigma, St. Louis, MO, USA) and 10 g of cholesterol powder was added to 5 kg of pulverized chow. Metabolizable energy from standard chow is 13.5 MJ/kg (9 energy% fat, 24 energy% protein, 67 energy% carbohydrates) whereas metabolizable energy from the CC diet is 15.7 MJ/kg (28 energy% fat, 19 energy% protein, 53 energy% carbohydrates). The experimental CC diet was maintained for 26 weeks. Reference SC diet mice and reference CC diet mice were analyzed at the age of 38 weeks. MDCO-216 SC diet and CC diet intervention groups were treated with 8 intraperitoneal administrations of 100 mg/kg (protein concentration) of MDCO-216 at an interval of 48 h each starting at the age of 38 weeks. Control buffer SC diet and CC diet mice were injected with the same volume of the buffer solution pH 7.4 containing mannitol 43.6 mM, sucrose 181 mM, NaH_2_PO_4_·2H_2_O 3.46 mM, and 8.43 mM Na_2_HPO_4_·7H_2_O (Figure 1). Endpoint analyses in the MDCO-216 and control buffer groups were performed at the age of 40 weeks. In the first experimental layer, mice were assigned for hemodynamic quantification and histochemical and immunohistochemical analysis. The second experimental layer consisted of mice that did not undergo perfusion fixation and that were used for quantification of tissue and organ weights and for quantification of protein expression levels.

Group assignment at the start of the study was performed randomly. No mice died during the study. No mice were excluded from the analysis. Endpoint analyses were performed by investigators who were blinded to the group allocation of the animal. Unblinding of animal numbers corresponding to specific allocation groups was performed at completion of measurements.

### 4.3. In Vivo Hemodynamic Measurements

Invasive hemodynamic measurements were performed before sacrifice following anesthesia induced by intraperitoneal administration of 1.2 g/kg urethane (Sigma). Measurements were performed using Millar’s Mikro-Tip^®^ ultra-miniature pressure-volume (PV) loop catheter PVR-1035 (1.0 French polyimide catheter), the MPVS Ultra Single Segment pressure-volume unit, and a PowerLab 16/35 data acquisition system (ADInstruments Ltd., Oxford, UK).

### 4.4. Lipoprotein and Murine Apo A-I Quantification in Plasma

Blood was obtained by puncture of the retro-orbital plexus. Anticoagulation was performed with 0.1 volume of 136 mmol/L trisodium citrate. Subsequently, plasma was immediately isolated by centrifugation at 1100× *g* for 10 min and stored at −20 °C. Plasma cholesterol levels were determined using Cholesterol Quantification kit from Sigma (Sigma). HDL and non-HDL lipoproteins were separated by ultracentrifugation as described before [25]. Murine apo A-I plasma levels were determined by enzyme-linked immunosorbent assay (ELISA) (Mabtech AB, Nacka Strand, Sweden). No cross-reaction with wild-type human apo A-I or human apo A-I_Milano_ was observed.

### 4.5. Quantification of Myocardial Protein Levels by Western Blot

Myocardial tissue samples isolated at the time of sacrifice were immediately frozen in liquid nitrogen and stored at −80 °C. Tissues were placed in lysing matrix tubes (QBiogene/MP Biomedicals, Solon, OH, USA), mixed with 1 mL of protein extraction buffer containing 10 mM imidazole, 300 mM sucrose, 1 mM dithiotreitol, 1mM sodium metabisulfite, 25 mM sodium fluoride, 5 mM sodium ethylenediaminetetraacetic acid, 5 mM sodium pyrophosphate, 0.3 mM phenylmethylsulfonyl fluoride, and a protease inhibitor cocktail (Roche Diagnostics Belgium, Vilvoorde, Belgium) (Lenaerts et al., 2013), and homogenized in the FastPrep24 instrument (MP Biomedicals). Protein concentration was quantified using the Pierce BCA Protein Assay kit (Pierce Biotechnology Inc., Rockford, IL, USA). Equal amounts of proteins were separated on 4–20% Tris-Glycine gradient gels (Bio-Rad Laboratories N.V., Temse, Belgium) and blotted onto polyvinylidene difluoride membranes (Bio-Rad Laboratories N.V.). Membranes were incubated with primary antibodies against acetyl-coenzyme A (acetyl-CoA) carboxylase (ACC), p-ACC (Ser79), transforming growth factor (TGF)-β1, glyceraldehyde 3-phosphate dehydrogenase (GAPDH) (all prior antibodies from Cell Signaling Technologies, Beverly, MA, USA), and osteopontin (Abcam, Cambridge, UK). Protein expression was detected with Super signal west pico chemilumninescent reagents (Thermo Scientific, Rockford, IL, USA) and quantified using Image lab TM Analyzer software (Bio-Rad laboratories N.V.). All protein levels were normalized to the GAPDH protein level.

### 4.6. Histological Analyses

Histological analyses were performed as described before [25]. After hemodynamic analyses, mice were perfused via the abdominal aorta with phosphate-buffered saline and hearts were arrested in diastole by KCl (100 μL; 0.1 mol/L), followed by perfusion fixation with 1% paraformaldehyde in phosphate-buffered saline. Thereafter, hearts were post-fixated overnight in 1% paraformaldehyde and embedded in paraffin. Cross-sections of 6 μm thickness at 130 μm spaced intervals were made extending from the apex to the basal part of the left ventricle. Comparative sections were analyzed for all histological analyses by using the same slide numbers (1 to 40 from apex to base) and cross-section numbers (1–10). 

To measure collagen content in the interstitium, Sirius Red staining was performed as described by Junqueira et al. [43]. Sirius Red polarisation microscopy on a Leica RBE microscope with KS300 software (Zeiss) was applied to quantify thick tightly packed mature collagen fibers as orange-red birefringent and loosely packed less cross-linked and immature collagen fibers as yellow-green birefringent. Collagen positive area was normalized to the LV wall area and was expressed as percentage. Any perivascular fibrosis was excluded from this analysis. Perivascular fibrosis was quantified as the ratio of the fibrosis area surrounding the vessel to the total vessel area. Two mid-ventricular sections were studied per animal [25].

Cardiomyocyte hypertrophy was analyzed on paraffin sections stained with rabbit anti-mouse laminin (Sigma; 1/50) by measuring the cardiomyocyte cross-sectional area (μm^2^) of at least 200 randomly selected cardiomyocytes in the LV myocardium. Capillary density in the myocardium was determined on CD31 stained sections using rat anti-mouse CD31 antibodies (BD; 1/500). Relative vascularity was calculated as the ratio of capillary density to cardiomyocyte density divided by the cardiomyocyte cross-sectional area [44] and is expressed in µm^−2^. Two mid-ventricular cross-sections were analyzed per mouse [45,46]. 

### 4.7. Exercise Treadmill Testing

A motor-driven treadmill (Treadmill Simplex II, Columbus Instruments, Columbus, OH, USA) was applied to evaluate exercise capacity in mice [47]. Mice were familiarized with running on a motorized treadmill for one week. To quantify endurance capacity, mice started running on a 10° incline at an initial speed of 10 m/min, which was increased by 1 m/min every minute until the mouse resides on the stimulus plate (pulse grill) for ≥5 s. At this point, the mouse was immediately removed from the treadmill. The total exercise time was recorded as the elapsed time to exhaustion (min) and was then converted to distance (m), which is the end-point. Mice were subjected to tail snip before and after exercise tolerance test for lactate analysis (EKF diagnostics, Penarth, Cardiff, UK).

### 4.8. Statistical Analysis

At the end of the study, data of all surviving mice were included in the analysis. Investigators who performed endpoint analyses were blinded to group allocation. Unblinding of animal numbers corresponding to specific allocation groups was performed at completion of measurements. 

Data are expressed as means ± standard error of the means (SEM). Minimally required sample size calculation (*n* = 13) for proving the effect of MDCO-216 on hemodynamic parameters in CC diet mice was based on a statistical power of 90%, a two-sided cut-off value of statistical significance of 0.05, a difference of main hemodynamic parameters at the population level of 20%, and a standard deviation at population level at 16% of the average of population means. Parameters between SC diet groups and respective CC diet groups were compared using Student’s *t* test. When indicated, a logarithmic transformation or a non-parametric Mann–Whitney test was performed. The assumption of Gaussian distribution was tested using the Kolmogorov–Smirnov method. Parameters between the three SC diet groups or between the three CC diet groups were compared by one-way analysis of variance followed by Tukey’s multiple comparisons groups using GraphPad Instat (GraphPad Software, San Diego, CA, USA). When the assumption of sampling from populations with identical standard deviations was not met, a logarithmic transformation was performed. When the assumption of sampling from populations with Gaussian distributions was not met, a Kruskal–Wallis test was performed followed by Dunn’s multiple comparisons post-test. Kaplan–Meier survival curves were analyzed by log-rank test using Prism4 (GraphPad Software). A two-sided p-value of less than 0.05 was considered statistically significant.

## Figures and Tables

**Table 1:** Overview of hemodynamic data in C57BL/6 mice fed standard chow and C57BL/6 mice fed the CC diet.

	Standard Chow (*n* = 15)	CC Diet (*n* = 22)
Heart rate (bpm)	588 ± 11	572 ± 9
P_max_ (mm Hg)	98.2 ± 1.2	81.8 ± 2.1 ****
P_es_ (mm Hg)	96.3 ± 1.0	74.7 ± 2.1 ****
dP/dt_max_ (mmHg/ms)	9.57 ± 0.67	8.00 ± 0.67 *
PRSW (mmHg)	82.7 ± 7.9	65.4 ± 2.9 *
E_es_ (mmHg/μL)	8.48 ± 0.61	3.97 ± 0.33 ****
P_min_ (mm Hg)	−0.528 ± 0.830	1.41 ± 0.36 *
P_ed_ (mm Hg)	1.90 ± 0.47	4.43 ± 0.30 ****
dP/dt_min_ (mmHg/ms)	−9.94 ± 0.71	−7.95 ± 0.38 *
Tau (ms)	5.39 ± 0.19	6.77 ± 0.26 ***
Slope EDPVR (mmHg/μL)	0.259 ± 0.039	0.765 ± 0.176 *
EDV (μL)	27.0 ± 2.0	23.3 ± 1.3
ESV (μL)	10.5 ± 1.3	10.4 ± 1.2
Stroke volume (μL)	16.5 ± 1.0	12.9 ± 0.9 *
Ejection fraction (%)	62.2 ± 2.8	56.5 ± 3.4
Cardiac output (ml/min)	9.67 ± 0.62	7.40 ± 0.55 *
Stroke work (mmHg·μL)	1290 ± 80	856 ± 74 ***
dV/dt_max_ (μL/s)	773 ± 66	579 ± 43 *
dV/dt_min_ (μL/s)	−821 ± 63	−607 ± 42 **
E_a_ (mmHg/μL)	6.33 ± 0.55	6.10 ± 0.37
E_a_/E_es_	0.809 ± 0.105	2.03 ± 0.38 ****

The 0.2% cholesterol 10% CC diet was initiated at 12 weeks of age. Hemodynamic measurements were performed at the age of 38 weeks, 26 weeks after the start of the diet. P_max_: maximum systolic pressure. Pes: end-systolic pressure. dP/dt_max_: peak rate of isovolumetric contraction. PRSW: preload recruitable stroke work. E_es_: end-systolic elastance. P_min_: minimum diastolic pressure. P_ed_: end-diastolic pressure. dP/dt_min_: peak rate of isovolumetric relaxation. Tau: time constant of isovolumetric relaxation. EDPVR: end diastolic pressure-volume relationship. EDV: end-diastolic volume. ESV: end-systolic volume. dV/dt_max_: peak filling rate. dV/dt_min_: peak emptying rate. E_a_: arterial elastance. E_a_/E_es_: ventriculo-arterial coupling ratio. All data are expressed as means ± SEM. *: *p* < 0.05; **: *p* < 0.01; ***: *p* < 0.001; ****: *p* < 0.0001.

**Table 2:** Total cholesterol, non-HDL cholesterol, and HDL cholesterol plasma levels in C57BL/6 mice at time of sacrifice.

	Reference SC Diet (*n* = 10)	Buffer SC Diet (*n* = 10)	MDCO-216 SC Diet (*n* = 10)	Reference CC Diet (*n* = 10)	Buffer CC Diet (*n* = 10)	MDCO-216 CC Diet (*n* = 10)
Total cholesterol	1.62 ± 0.08	1.61 ±0.08	1.64 ±0.10	1.67 ± 0.08	1.69 ±0.09	1.61 ±0.10
Non-HDL cholesterol	0.325 ± 0.048	0.365 ± 0.047	0.389 ± 0.052	0.409 ± 0.060	0.416 ± 0.040	0.331 ± 0.052
HDL cholesterol	1.29 ± 0.07	1.24 ± 0.10	1.25 ± 0.12	1.26 ±0.06	1.27 ± 0.06	1.28 ± 0.12

Data are expressed in mmol/L and represent means ± SEM. Reference mice were sacrificed at the age of 38 weeks whereas buffer mice and MDCO-216 mice were sacrificed at the age of 40 weeks.

**Table 3:** Overview of hemodynamic data in C57BL/6 mice and in intervention C57BL/6 mice fed standard chow.

	Reference SC Diet (*n* = 15)	Buffer SC Diet (*n* = 11)	MDCO-216 SC Diet (*n* = 10)	Reference CC Diet (*n* = 22)	Buffer CC Diet (*n* = 13)	MDCO-216 CC Diet (*n* = 14)
Heart rate (bpm)	588 ± 11	584 ± 22	594 ± 13	572 ± 9	579 ± 20	587 ± 19
P_max_ (mm Hg)	98.2 ± 1.2	96.6 ±3.7	100 ± 1	81.8 ± 2.1 ^!!!!^	83.3 ± 4.2 ^!^	97.7 ± 3.7 ^§§^*
P_es_ (mm Hg)	96.3 ± 1.0	93.0 ±3.6	95.5 ± 1.3	74.7 ± 2.1 ^!!!!^	80.5 ± 4.0 ^!^	93.3 ± 3.2 ^§§§^*
dP/dt_max_ (mmHg/ms)	9.57 ± 0.67	10.3 ± 0.4	10.2 ± 0.5	8.00 ± 0.67 ^!^	8.22 ± 0.31 ^!!!^	12.2 ± 0.6 ^§§§^**
PRSW (mmHg)	82.7 ± 7.9	80.0 ± 7.3	79.5 ± 4.7	65.4 ± 2.9 ^!^	64.0 ± 2.4 ^!^	77.6 ± 4.2 ^§^*
E_es_ (mmHg/μL)	8.48 ± 0.61	8.23 ± 0.74	8.23 ± 0.74	3.97 ± 0.33 ^!!!!^	4.58 ± 0.42 ^!!!^	8.31 ± 0.63 ^§§^***
P_min_ (mm Hg)	−0.528 ± 0.830	1.58 ± 0.41	0.0839 ± 0.648	1.41 ± 0.36 ^!^	2.38 ± 0.36	0.601 ± 0.534*
P_ed_ (mm Hg)	1.90 ± 0.47	2.64 ± 0.51	1.92 ± 0.54	4.43 ± 0.30 ^!!!!^	4.30 ± 0.34 ^!^	3.80 ± 0.47
dP/dt_min_ (mmHg/ms)	−9.94 ± 0.71	−9.98 ± 0.31	−10.3 ± 0.5	−7.95 ± 0.38 ^!^	−8.01 ± 0.33 ^!!!^	−9.99 ± 0.32 ^§§§^**
Tau (ms)	5.39 ± 0.19	5.38 ± 0.25	5.05 ± 0.27	6.77 ± 0.26 ^!!!^	7.67 ± 0.44 ^!!!^	5.23 ± 0.24 ^§§§^***
Slope EDPVR (mmHg/μL)	0.259 ± 0.039	0.314 ± 0.052	0.237 ± 0.035	0.765 ± 0.176 ^!^	0.617 ± 0.138	0.336 ± 0.061
EDV (μL)	27.0 ± 2.0	25.0 ±0.8	26.2 ± 1.2	23.3 ± 1.3	24.5 ± 1.2	26.8 ± 1.4
ESV (μL)	10.5 ± 1.3	9.56 ± 0.41	9.58 ± 1.29	10.4 ± 1.2	11.1 ± 0.9	9.76 ± 1.11
Stroke volume (μL)	16.5 ± 1.0	15.4 ± 0.7	16.6 ± 0.8	12.9 ± 0.9 ^!^	13.4 ± 0.6 ^!^	17.0 ± 0.9 ^§§^*
Ejection fraction (%)	62.2 ± 2.8	61.5 ±1.6	64.3 ± 3.8	56.5 ± 3.4	55.3 ± 2.0	64.4 ± 2.9*
Cardiac output (ml/min)	9.67 ± 0.62	9.00 ± 0.56	9.84 ± 0.49	7.40 ± 0.55 ^!^	7.84 ± 0.55	10.0 ± 0.7 ^§§^*
Stroke work (mmHg·μL)	1290 ± 80	1180 ± 80	1330 ± 70	856 ± 74 ^!!!^	909 ± 78 ^!^	1330 ± 100 ^§§§^**
dV/dt_max_ (μL/s)	773 ± 66	725 ± 44	778 ± 87	579 ± 43 ^!^	536 ± 51 ^!^	774 ± 90 *
dV/dt_min_ (μL/s)	−821 ± 63	−766 ± 31	−829 ± 64	−607 ± 42 ^!!^	−538 ± 67 ^!!^	−717 ± 80
E_a_ (mmHg/μL)	6.33 ± 0.55	6.15 ± 0.34	5.89 ± 0.33	6.10 ± 0.37	6.04 ± 0.26	5.70 ± 0.36
E_a_/E_es_	0.809 ± 0.105	0.820 ± 0.091	0.752 ± 0.078	2.03 ± 0.38 ^!!!!^	1.51 ± 0.21 ^!!^	0.740 ± 0.074 ^§§§^***

Hemodynamic measurements were performed at the age of 38 weeks in the standard chow group. Eight intraperitoneal injections of reconstituted HDL_Milano_ (MDCO-216) (100 mg/kg) or of an equivalent volume of control buffer were executed with a 48-h interval starting at the age of 38 weeks. Analysis in the buffer and in the HDL_Milano_ group was performed at 38 weeks plus 15 days. P_max_: maximum systolic pressure. P_es_: end-systolic pressure. dP/dt_max_: peak rate of isovolumetric contraction. PRSW: preload recruitable stroke work. E_es_: end-systolic elastance. P_min_: minimum diastolic pressure. P_ed_: end-diastolic pressure. dP/dt_min_: peak rate of isovolumetric relaxation. Tau: time constant of isovolumetric relaxation. EDPVR: end diastolic pressure-volume relationship. EDV: end-diastolic volume. ESV: end-systolic volume. dV/dt_max_: peak filling rate. dV/dt_min_: peak emptying rate. E_a_: arterial elastance. E_a_/E_es_: ventriculo-arterial coupling ratio. All data are expressed as means ± SEM. ^!^: *p* < 0.05; ^!!^: *p* < 0.01; ^!!!^: *p* < 0.001; ^!!!^: *p* < 0.0001 versus respective SC diet group. ^§^: *p* < 0.05; ^§§^: *p* < 0.01; ^§§§^: *p* < 0.001 versus CC diet reference. *: *p* < 0.05; **: *p* < 0.01; ***: *p* < 0.001 versus CC diet buffer.

## Abbreviations

HFpEF	Heart failure with preserved ejection fraction
HFrEF	Heart failure with reduced ejection fraction
CC	0.2% cholesterol 10% coconut oil
SC	standard chow
apo	apolipoprotein
HDL	high-density lipoprotein
LV	left ventricle
POPC	1-palmitoyl-2-oleoyl-sn-glycero-3-phosphatidylcholine
TAC	transverse aortic constriction
MPVS	Millar Pressure-Volume (PV) Loop System
